# Renal Effects of Cannabigerol—Regulation of Lipid Metabolism in the Early Stage of Metabolic Kidney Disorders Induced by High-Fat High-Sucrose Diet

**DOI:** 10.3390/nu18132063

**Published:** 2026-06-24

**Authors:** Klaudia Sztolsztener, Tomasz Michał Tomczyk, Irena Kasacka, Ewa Harasim-Symbor, Adrian Chabowski, Karolina Konstantynowicz-Nowicka

**Affiliations:** 1 Department of Physiology, Medical University of Bialystok, Mickiewicz 2C Str., 15-222 Bialystok, Poland; tomasz.michal.tomczyk@gmail.com (T.M.T.); ewa.harasim-symbor@umb.edu.pl (E.H.-S.); adrian.chabowski@umb.edu.pl (A.C.); karolina.konstantynowicz-nowicka@umb.edu.pl (K.K.-N.); 2 Department of Histology and Cytophysiology, Medical University of Bialystok, Mickiewicz 2C Str., 15-222 Bialystok, Poland; irena.kasacka@umb.edu.pl

**Keywords:** lipid metabolism, fatty acid, cannabigerol, high-fat high-sucrose diet, kidney disorders, metabolic kidney diseases, kidney tissue

## Abstract

Background: Kidney disorders are strongly related to metabolic disturbances, including obesity and type 2 diabetes. Excessive intake of sugar and saturated fats promotes lipid accumulation, cellular energy issues and inflammatory responses. Cannabigerol (CBG), a non-psychotropic phytocannabinoid, has recently gained attention for its metabolic, anti-inflammatory and potential protective properties. Methods: The present study investigated the effect of two weeks of CBG administration (last 14 days of the experiment) on fatty acid (FA) composition, FA metabolic pathways and FA transporters in rats subjected to a high-fat high-sucrose diet (HFHS) for 6 weeks. Male Wistar rats were divided into four groups: Control, CBG, HFHS, and HFHS+CBG. Kidney tissue and urine samples were analyzed by gas–liquid chromatography (GLC) for lipid fractions and FA profiles, while protein expression of FA transporters and metabolic enzymes was assessed by immunoblotting. Polysaccharides and collagen fibers were visualized using Periodic Acid-Schiff (PAS) and AZAN staining, respectively. ELISA and colorimetric kits were used to measure urinary albumin and creatinine contents. Results: HFHS feeding altered renal lipid homeostasis, increasing saturated and monounsaturated fatty acids (SFA and MUFA, respectively) levels and affecting desaturation and elongation ratios. CBG supplementation affected renal lipid metabolism by lowering triacylglycerol (TAG) accumulation, restoring polyunsaturated fatty acids (PUFA) in phospholipid (PL) and altering FA ratios, suggesting an improvement in lipid balance. CBG also increased the expression of carnitine palmitoyltransferase 1 (CPT1) and lipoprotein lipase (LPL) and decreased the expression of stearoyl-CoA desaturase 1 (SCD1) and fatty acid synthase (FAS), suggesting a shift toward enhanced FA oxidation and reduced lipogenesis. Conclusions: Overall, CBG exerted good effects on renal lipid metabolism and may mitigate early lipid-mediated injury associated with metabolic kidney disorders.

## 1. Introduction

For the last 30 years, we have observed a rapid increase in the incidence of metabolic diseases in the general population. According to the World Health Organization (WHO), in 2022 almost half of the over-25-year-old population all around the world was stricken by overweight or obesity. This puts them at significant risk of developing chronic illnesses, including type 2 diabetes mellitus (T2DM), hypertension and hypercholesterolemia, further escalating health issues. Unsatisfactory predictions are held, as the expected number of people over 25 years old being overweight or obese is projected to rise from 2.11 billion to 3.8 billion in 2050 [1,2,3]. Along with this surge, morbidities will arise. One of the most common consequences, but also intractable, is metabolic kidney disease. Developing in approximately 40% of T2DM patients, this microvascular complication is considered to be the main cause of chronic kidney disease (CKD) and end-stage renal disease (ESRD) [4]. Following the confirmation of diagnosis of metabolic kidney disease, according to the Renal Pathology Society, it can be categorized into four classes according to the increasing advancement of the disease, depending on the histopathological examination [5]. An important role in the pathogenesis of those visual alterations is performed by heterogeneous groups of lipids and their derivatives, as their physiological function varies from being the most abundant constituent in the cell membrane to producing energy or exhibiting inflammatory responses [6]. However, dysregulation of fatty acid (FA) content is often regarded as an implication of kidney disorders; it is also considered an important factor that can lead to proinflammatory and profibrotic consequences. Lipid disorder, caused by excess accumulation of lipids in cells of non-adipose tissue, is referred to as lipotoxicity, which nowadays is widely influenced by the Western diet [7,8]. A high-fat diet (HFD) impairs mitochondrial fatty acid ß-oxidation through a reduction in phosphorylation of essential factors, including AMP-activated kinase (AMPK) and acetyl-CoA carboxylase (ACC), consecutively leading to oxidative stress and cytotoxicity [9]. Other possible mechanisms involve a direct outcome of excessive lipid peroxidation—ferroptosis and malfunction of the autophagy-lysosomal system [10,11]. It is predominantly determined by excessive tissue uptake of saturated fatty acids (SFA), with palmitic acid (PA; C16:0) being the most dietary prevalent SFA and stearic acid (C18:0), myristic acid (C14:0), and lauric acid (C14:0) following [12]. Contrary to the toxic impact of the abovementioned alterations, polyunsaturated fatty acids (PUFA) and monounsaturated fatty acids (MUFA) benefit renal function. Unsaturated fatty acids (UFA) counteract the development of lipotoxicity due to metabolic regulation. Moreover, elevation in the levels of PUFA and MUFA is associated with reduced levels of serum inflammation markers, such as interleukin 6 (IL-6) or tumor necrosis factor α (TNF-α) [13], which may increase the risk of kidney stones and other complications. Recent findings also indicate that the relationship between intestinal microbiota and their byproducts is crucial for managing overall lipid and energy metabolism. For example, some data show that modulation of gut microbiota composition and intestinal-related metabolites can affect both the homeostasis and inflammatory responses [14,15]. According to these findings, renal lipid metabolism may be regulated by mechanisms involving microbiota-derived metabolic signaling. Nonetheless, this idea is still a hypothesis and needs to be confirmed through direct experiments in upcoming research.

Current therapy for metabolic kidney disease is based on profoundly overlooked drugs that support symptomatic and casual treatment; however, it does not comprehensively eliminate the risk of progression and the demand for appropriate adjustments is substantial [10,16]. In recent years, we have observed a surge of interest in cannabinoid compounds related to well-established and potential utilization in medical practice. The absence of psychomimetic effects alongside diverse health benefits has increasingly drawn researchers’ attention to the phytocannabinoid (*Cannabis sativa L*.) precursor of Δ9-tetrahydrocannabinol (Δ9-THC) and cannabidiol (CBD)—cannabigerol (CBG) [17,18]. Cannabinoids exert their activity through the endocannabinoidome (eCBome) system. Furthermore, a lot of evidence points out the function of the eCBome system in renal homeostasis. Cannabigerol is identified as a partial agonist to cannabinoid receptors 1 and 2 (CB_1_ and CB_2_) and also G protein-coupled receptors (GPCR) considered at present as the primary receptors of the eCBome system in human physiology. Those two receptors have been shown to display opposite impacts on renal function and structure in diabetic nephropathy (DN) and obesity-related kidney disease. Inactivation of CB_1_ and activation of CB_2_ reduced the expression of inflammatory and fibrotic markers, simultaneously reducing albuminuria [19]. Some studies reveal promising properties as a result of reduced renal lipid accumulation, whereas others indicate accumulation of lipid droplets in renal tubular epithelial cells (TECs) and fibrosis associated with lipotoxicity due to CB_2_ activation [20,21].

This data, combined with its well-known therapeutic effects, implies its possible utilization in obesity-related kidney disease management [19]. According to the gathered resources, our study’s primary focus is renal lipid metabolism with direct emphasis on FA synthesis and oxidation in rats fed with a high-fat high-sucrose diet (HFHS). Moreover, we have investigated the expression of key enzymes involved in the regulation of the mentioned pathways. Furthermore, our investigation will include the count of desaturation and elongation indexes with the de novo synthesis pathway of certain selected FA.

## 2. Materials and Methods

### 2.1. Experimental Model

The experiment was conducted on 40 male Wistar rats with 70–100 g of body weight at the beginning. All animals were kept in standard holding conditions, specifically unlimited access to water and standard rodent chow, 22 ± 2 °C air temperature, 55 ± 10% air humidity and a reverse 12 h/12 h light/dark cycle. After one week of acclimatization period, rats were randomly divided into four groups (n = 10) in such a way (1) Control group—rats fed with a standard diet (Labofeed B, Kcynia, Poland; calories distribution: 67% carbohydrate, 25% protein and 8% fat) with plain water to drink, (2) CBG group—rats fed with a standard diet with plain water to drink and the supplementation of cannabigerol (CBG; TargetMol, Wellesley Hills, MA, USA), (3) HFHS group—rats fed with a high-fat diet (HFD; Research Diet, New Brunswick, NJ, USA; calories distribution: 20% carbohydrate, 20% protein and 60% fat) with 20% *w*/*v* solution of sucrose (Sigma Aldrich, Saint Louis, MO, USA) to drink, (4) HFHS+CBG group—rats fed with an HFD with 20% *w*/*v* sucrose solution to drink and the supplementation of cannabigerol. The CBG solution was prepared by dissolving powder in sesame seed oil (Oleofarm, Wroclaw, Poland) at a concentration of 30 mg/kg of body weight [22,23], which was monitored every week during all six weeks of the experiment. Rats from the Control and HFHS groups received sesame seed oil. The CBG solution and sesame seed oil were applied by intragastrical gavage once a day during the last 14 days (5th and 6th weeks) of the experiment. After 6 weeks of experimental feeding, rats were anesthetized by intraperitoneal administration of phenobarbital at a concentration of 80 mg/kg of body weight. Urine samples were collected into fresh Eppendorf tubes. Kidney tissues were excised and cut in half. Samples were frozen in the temperature of liquid nitrogen and stored at −80 °C for further measurements.

All experimental procedures were conducted according to the national and institutional guidelines for the care and use of laboratory animals, supported by the Local Ethical Committee for Animal Experiments in Olsztyn (approval No. 19/2022).

### 2.2. Histological Staining

The kidneys of the rats were taken from each animal and immediately fixed in 10% buffered formalin and processed routinely for embedding in paraffin. The paraffin blocks were cut into 4 µm sections and attached to positively charged glass slides (Superfrost Plus; Menzel Gläser, Braunschweig, Germany). Paraffin-embedded sections were deparaffinized in xylene and hydrated in a series of ethanol (99.8%, 95% and 70%).

For periodic acid-Schiff staining, sections were oxidized in 0.5% periodic acid for 5 min, rinsed in distilled water and stained with Schiff reagent for 15 min. Then, prepared slides were washed in lukewarm tap water for 5 min until a dark pink color developed. Following counterstaining in Mayer’s hematoxylin for 1 min, the slides were washed under running tap water.

For azan staining, sections were stained with Azocarmine G solution (preheated at 50 °C) for 60 min, followed by an additional 60 min at RT. Next, the differentiating process in an aniline-alcohol solution was carried out until the nuclei stood out sharply. After rinsing in acetic alcohol for 2 min and washing in running tap water, sections were treated with 5% phosphomolybdic acid for 180 min at RT, rinsed in distilled water, and stained with an aniline blue-orange G mixture for 30 min. Final differentiation was performed in 100% ethanol until the blue and red colors were clearly distinguishable.

Lastly, all slides were dehydrated (70%, 80%, 95%, 99.8% ethanol and xylene) and mounted using a synthetic mounting medium. Staining results were evaluated using an Olympus BX43 microscope with an Olympus DP12 camera and analyzed in a blind slide reading procedure. Histological quantification was performed on kidneys obtained from animals included in the study (n = 6). For each animal, 3 sections were evaluated at ×200 magnification. Quantitative assessment was performed in the glomerular and tubulointerstitial compartments. PAS-stained sections were used to assess polysaccharides and glycolipid deposition, whereas AZAN-stained sections were used to quantify collagen deposition. Digital images were quantitatively analyzed using ImageJ software (National Institutes of Health, Bethesda, MD, USA).

### 2.3. Gas–Liquid Chromatography

Gas–liquid chromatography (GLC) method was used to measure the content of total lipid fractions (triacylglycerol (TAG), diacylglycerol (DAG), free fatty acid (FFA), phospholipid (PL)) and the composition of fatty acid (FA) in each mentioned fraction (C14:0—myristic acid, C16:0—palmitic acid, C18:0—stearic acid, C20:0—arachidic acid, C22:0—behenic acid, C24:0—lignoceric acid, C16:1—palmitoleic acid, C18:1—oleic acid, C24:1—nervonic acid, C18:2—linoleic acid, C18:3—linolenic acid, C20:4—arachidonic acid, C20:5—eicosapentaenoic acid, C22:6—docosahexaenoic acid) in the kidney tissue and urine samples. Based on measured FA composition, saturated fatty acids (SFA; the sum of C14:0, C16:0, C18:0, C20:0, C22:0, C24:0), monounsaturated fatty acids (MUFA; the sum of C16:1, C18:1, C24:1) and polyunsaturated fatty acids (PUFA; the sum of C18:2, C18:3, C20:4, C20:5, C22:6) were calculated in all lipid pools. Moreover, in the kidney tissue, the estimation of selected FA ratios was also conducted. There were elongation ratios (C18:0/C16:0, C20:0/C18:0, C22:0/C20:0, C24:0/C22:0), desaturation ratios (C18:1/C18:0, C24:1/C24:0) and de novo lipogenesis index (C16:0/C18:2).

In brief, lipids were extracted from kidney tissues and urine samples in a chloroform/ethanol mixture (2:1 ratio, *v*/*v*) in accordance with the Folch et al. method [24] in the presence of butylated hydroxytoluene and heptadecanoic acid as an internal standard. After overnight incubation and addition of water, samples were centrifuged at 845× *g*, 4 °C for 10 min and the bottom layer was moved to fresh tubes. Obtained extracts were developed on silica plates during thin-layer chromatography (TLC) in the presence of resolving solution (heptane/isopropyl ether/and hexane (60:40:3, *v/v/v*)). Divided fractions, i.e., TAG, DAG, FFA and PL, were visualized under UV light and collected by scratching into tubes. Separated fractions were evaluated in diethyl ether/hexane solution (1:1, *v*/*v*) and chloroform/methanol/water solution (5:5:1, *v*/*v*/*v*). Following evaporation of the obtained organic phase in a nitrogen stream, samples were transmethylated in accordance with the method described by Christie [25]. For that purpose, a mixture of diethyl ether and methyl acetate was added to the isolated TAG and PL fractions and gently mixed. Then, lipids were reacted with 1M sodium methoxide in methanol at RT for 10 min, after which the reaction was stopped by the addition of oxalic acid in diethyl ether (saturated solution) and gently mixed, following the next evaporation of the obtained solvent in a nitrogen stream. In another fraction, DAG, the methylation process was conducted by the addition of boron trifluoride in methanol and incubation at 100 °C for 10 min. After that, pentane was used to extract fatty acid methyl esters (FAME), which in the next step was evaporated in a nitrogen stream. All lipid fractions were then dissolved in hexane and analyzed on a gas chromatograph (Hewlett-Packard 5890 Series; Agilent Technologies, Santa Clara, CA, USA) equipped with a flame ionization detector and a capillary column (HP-INNOWax GLC column; Agilent Technologies, Santa Clara, CA, USA). Based on a standard curve for each FA in each lipid fraction for kidney tissue and urine samples, the level of total lipid fractions, SFA, MUFA, PUFA and FA composition was calculated and expressed in nanomoles per gram of tissue or nanomoles per milliliter of urine.

### 2.4. Immunoblotting

Immunoblotting method was used to measure the expression of fatty acid transporters, i.e., fatty acid transporter 1 (FATP1; Santa Cruz Biotechnology, Inc., Dallas, TX, USA), fatty acid transporter 2 (FATP2; Santa Cruz Biotechnology, Inc., Dallas, TX, USA), fatty acid transporter 4 (FATP4; Abcam, Cambridge, UK), fatty acid translocase (CD36; Santa Cruz Biotechnology, Inc., Dallas, TX, USA), plasma membrane fatty acid-binding protein (FABPpm; Abcam, Cambridge, UK), and proteins regulating fatty acid metabolism, i.e., stearoyl-CoA desaturase 1 (SCD1; Abcam, Cambridge, UK), fatty acid synthase (FAS; Cell Signaling Technology, Inc., Danvers, MA, USA), carnitine palmitoyltransferase 1 (CPT1; Santa Cruz Biotechnology, Inc., Dallas, TX, USA), sterol regulatory element-binding transcription factor 1 (SREBP1; Santa Cruz Biotechnology, Inc., Dallas, TX, USA), lipoprotein lipase (LPL; Santa Cruz Biotechnology, Inc., Dallas, TX, USA). The dilution and catalog number of used antibodies are listed in Table 1.

In brief, the kidney tissue was homogenized in the presence of ice-cold radioimmunoprecipitation assay (RIPA) buffer with the addition of phosphatase and protease inhibitors (Roche Diagnostics, GmbH, Mannheim, Germany). Following incubation at 4 °C for 45 min, samples were centrifuged at 1000× *g*, 4 °C for 30 min. Supernatants were moved into new tubes and applied to determine the protein concentration using the bicinchoninic acid (BCA) protein assay kit and bovine serum albumin (BSA) as a standard. Samples were restored in Laemmli buffer (Bio-Rad, Hercules, CA, USA) to obtain 30 µg of protein and loaded onto AnykD Criterion TGX Stain-Free Precast Gels (cat. no: 5678125; Bio-Rad, Hercules, CA, USA) followed by electrophoresis process. Then, proteins were transferred onto nitrocellulose or polyvinylidene fluoride (PVDF) membranes for wet or semi-dry transfers, respectively. Non-specific bands were blocked in 5% BSA or non-fat dry milk in Tris-buffered saline with Tween detergent (TBS-T). Membranes were overnight immunoblotted with an appropriate primary antibody. After that, membranes were washed with TBS-T and incubated with a horseradish peroxidase (HRP)-conjugated secondary antibody. Following washing with TBS-T, protein bands were visualized using a Clarity Western ECL Substrate (Bio-Rad, Hercules, CA, USA). Densitometric determination was conducted using a ChemiDoc visualization system (Image Laboratory Software; Bio-Rad, Hercules, CA, USA). The expression of proteins was normalized to the total protein (blot images after the transfer process), which was set as 100% for results from the Control group.

**Table 1:** List of primary antibodies used for immunoblotting.

Target Protein	Full Name	Source (Clone Number)	Dilution	Catalog Number	Manufacturer
FATP1	fatty acid transport protein 1	Rabbit (polyclonal)	1:500	sc-25541	Santa Cruz Biotechnology, Inc., Dallas, TX, USA
FATP2	fatty acid transport protein 2	Mouse (polyclonal)	1:200	sc-393906	Santa Cruz Biotechnology, Inc., Dallas, TX, USA
FATP4	fatty acid transport protein 4	Rabbit (monoclonal)	1:500	ab200353	Abcam, Cambridge, UK
CD36	fatty acid translocase	Mouse (monoclonal)	1:500	sc-7309	Santa Cruz Biotechnology, Inc., Dallas, TX, USA
FABPpm	membrane-associated fatty acid binding protein	Rabbit (monoclonal)	1:5000	ab153924	Abcam, Cambridge, UK
SCD1	stearoyl-CoA desaturase 1	Rabbit (monoclonal)	1:1000	ab236868	Abcam, Cambridge, UK
CPT1	carnitine palmitoyltransferase 1	Mouse (monoclonal)	1:500	sc-393070	Santa Cruz Biotechnology, Inc., Dallas, TX, USA
SREBP1	sterol regulatory element-binding transcription factor 1	Mouse (monoclonal)	1:500	sc-365513	Santa Cruz Biotechnology, Inc., Dallas, TX, USA
LPL	lipoprotein lipase	Mouse (monoclonal)	1:500	sc-373759	Santa Cruz Biotechnology, Inc., Dallas, TX, USA
FAS	fatty acid synthase	Rabbit (monoclonal)	1:1000	3180s	Cell Signaling Technology, Inc., Danvers, MA, USA
LXRα	liver X receptor alpha	Goat (polyclonal)	1:500	sc-1202	Santa Cruz Biotechnology, Inc., Dallas, TX, USA
LXRβ	liver X receptor beta	Mouse (monoclonal)	1:500	sc-34343	Santa Cruz Biotechnology, Inc., Dallas, TX, USA
PPARγ	peroxisome proliferator-activated receptor gamma	Mouse (monoclonal)	1:500	sc-7273	Santa Cruz Biotechnology, Inc., Dallas, TX, USA
GPx-1/2	glutathione peroxidase 1/2	Mouse (monoclonal)	1:500	sc-133160	Santa Cruz Biotechnology, Inc., Dallas, TX, USA

### 2.5. Enzyme-Linked Immunosorbent Assay and Colorimetric Assay

An enzyme-linked immunosorbent assay kit for urinary albumin level (Antibodies.com LLC, St. Louis, MO, USA) and a colorimetric assay kit for creatinine level (Elabscience, Houston, TX, USA) were used in the present measurements.

Before the albumin assay, samples were centrifuged at 1000× *g*, 4 °C for 20 min. Samples and standards were added to microplate wells and incubated. Following washing, a biotinylated detection antibody working solution was added to each well. After the next incubation, wells were washed and the HRP-streptavidin conjugate working solution was added to each well. Subsequently, the washing step was repeated and the reaction was developed by the addition of TMB substrate. The enzymatic reaction was stopped by the addition of stop solution, and the absorbance was measured at 450 nm using the Synergy H1 Hybrid Reader (BioTek Instruments, Winooski, VT, USA). The concentration of urinary albumin was calculated based on standard curves and expressed in nanograms per milliliter of urine.

In the case of colorimetric assay of creatinine concentration, urine samples were centrifuged at 1000× *g*, 4 °C for 20 min. The supernatants were diluted 50 times in 0.9% NaCl. Diluted samples and standard solutions were added to the proper wells. Next, enzymatic solution A was added to each well, followed by the plate incubation. Enzymatic solution B was added and the plate was incubated for 2 min. After that the absorbance was read at 515 nm using the Synergy H1 Hybrid Reader (BioTek Instruments, Winooski, VT, USA). The incubation was continued for the next 8 min and the absorbance reading at 515 nm was repeated. The concentration of creatinine was calculated according to the manufacturer’s protocol based on the standard curve and expressed in milligrams per deciliter of urine.

### 2.6. Statistical Analysis

Statistical analysis of our results was performed using GraphPad Prism 8.0 (San Diego, CA, USA). Data were presented as mean ± standard deviation (SD) and based on ten independent measurements (n = 10), except for Western blot and histological staining methods (n = 6). First, normal distribution and homogeneity of variance were assessed using the Shapiro–Wilk test and Bartlett test, respectively. Statistical differences were evaluated using one-way ANOVA following the Tukey test. The Mann–Whitney U test was used to test the assumptions for parametric analysis. Significant differences were set as *p* < 0.05. They were marked as * *p* < 0.05—significant difference between CBG, HFHS and HFHS+CBG groups vs. Control group and # *p* < 0.05—significant difference between HFHS+CBG group vs. HFHS group.

## 3. Results

### 3.1. Cannabigerol Influence on the Content of Total Lipid Fractions and Fatty Acid Composition in Each of Them in the Kidney Tissue of Rats Fed with a Standard Diet or a High-Fat High-Sucrose Diet

In the kidney tissue, cannabigerol (CBG) supplementation alone reduced the level of total triacylglycerol (TAG) and saturated fatty acids (SFA) in TAG pool (TAG–CBG: −66.3%, *p* < 0.05, Table 2; SFA–CBG: −55.9%, *p* < 0.05, Table 2) compared to the Control group. In the same experimental group, we observed a decrease in the level of mono- and polyunsaturated fatty acids (MUFA and PUFA) in diacylglycerol (DAG) (MUFA–CBG: −27.4%, *p* < 0.05, Table 2; PUFA–CBG: −22.7%, *p* < 0.05, Table 2). In free fatty acid (FFA) fraction, MUFA level was reduced in all experimental groups (CBG: −48.3%, HFHS: −53.0%, HFHS+CBG: −38.4%, *p* < 0.05, Table 2) more than in the Control rats. Opposite, FFA’s PUFA content was elevated in the CBG and HFHS+CBG groups (CBG: +136.5%, HFHS+CBG: +67.5%, *p* < 0.05, Table 2) compared to the Control and HFHS groups, respectively. Among phospholipid (PL) fraction, MUFA level was enhanced in the HFHS+CBG group (HFHS+CBG: +13.4% and +8.1%, *p* < 0.05, vs. Control and HFHS groups, respectively, Table 2). Another, PUFA level in PL was diminished in HFHS-fed rats (HFHS: −14.2%, HFHS+CBG: +18.0%, *p* < 0.05, Table 2) in relation to the Control and HFHS groups, respectively.

In the CBG group, in TAG fraction, we observed a decrease in the content of C14:0, C16:0, C18:0, C16:1, C18:3 (CBG: −48.6%, −55.5%, −48.5%, −53.9%, −38.9%, respectively, *p* < 0.05, Table S1) compared to the Control group. In the HFHS group, TAG’s level of C14:0, C16:1 was declined (HFHS: −27.8%, −30.2%, respectively, *p* < 0.05, Table S1) and TAG’s level of C18:1 was enhanced (HFHS: +57.1%, *p* < 0.05, Table S1) in comparison with the Control group. In the same lipid fraction, we also noticed a reduction in the level of C18:0, C16:1 in rats fed with an HFHS and CBG (HFHS+CBG: −63.9% and −72.2%, −39.5%, respectively, *p* < 0.05, Table S1) in relation to the Control and HFHS groups, respectively.

In the CBG group, in DAG fraction, the level of C22:0 was enhanced (CBG: +79.5%, *p* < 0.05, Table S2) and the levels of C16:1, C18:1, C18:2, C18:3 were declined (CBG: −29.3%, −25.5%, −25.6%, −32.2%, respectively, *p* < 0.05, Table S2) as opposed to the Control group. Moreover, in HFHS-fed rats, an enhancement in DAG’s content of C22:0, C18:1, C24:1 was observed (HFHS: +120.9%, +56.9%, +56.5%, respectively, *p* < 0.05, vs. Control group, Table S2). The supplementation of cannabigerol to rats subjected to an HFHS diet caused increases in C22:0, C24:1, C18:3 levels (HFHS+CBG: +119.4%, +65.6%, +31.9%, respectively, *p* < 0.05, vs. Control group, Table S2) and a decrease in C16:1 level (HFHS+CBG: −56.8% and +45.4%, *p* < 0.05, vs. Control and HFHS groups, respectively, Table S2) in DAG fraction.

In the FFA fraction, rats from the CBG group had a higher C14:0 level (CBG: +40.2%, *p* < 0.05, Table S3) and lower C20:0, C22:0, C24:0, C16:1, C18:1 levels (CBG: −48.0%, −54.2%, −49.1%, −35.9%, −51.7%, respectively, *p* < 0.05, Table S3) compared to the Control group. Similarly, in FFA, rats that received an HFHS diet only had an elevated content of C18:0, C20:0, C22:0, C24:0, C24:1 (HFHS: +44.5%, +49.5%, +93.0%, +69.0%, +85.8%, respectively, *p* < 0.05, Table S3) and a reduced content of C14:0, C16:1, C18:1, C18:2 (HFHS: −45.5%, −73.0%, −51.7%, −62.0%, respectively, *p* < 0.05, Table S3) as opposed to the Control group. In the HFHS+CBG group, FFA’s concentration of C14:0, C20:0, C22:0, C24:0, C16:1, C24:1, C18:3 were reduced (HFHS: −50.6%, −46.4%, −55.6%, −41.4% and −65.3%, −71.8%, −44.3%, −37.6%, *p* < 0.05, respectively, Table S3) and FFA’s concentration of C16:0, C18:2 were enhanced (HFHS: +36.5% and +47.1%, +93.6%, *p* < 0.05, Table S3) in comparison with the Control and/or HFHS groups.

In the HFHS group, in phospholipid fraction, we noticed increases in C14:0, C18:1 levels (HFHS: +28.1%, +11.5%, respectively, *p* < 0.05, Table S4) and decreases in C16:1, C18:2, C18:3 levels (HFHS: −43.5%, −19.3%, −34.5%, respectively, *p* < 0.05, Table S4) than in the Control group. In the same lipid fraction, rats that received an HFHS diet and CBG had higher C20:0, C18:1, C18:3 levels (HFHS+CBG: +22.1%, +21.9% and +9.3%, +18.0%, respectively, *p* < 0.05, Table S4) and lowered C24:0, C16:1 levels (HFHS+CBG: −41.0% and −35.7%, −40.4%, respectively, *p* < 0.05, Table S4) compared to the Control and/or HFHS groups.

### 3.2. Cannabigerol Influence on the Content of Total Lipid Fractions and Fatty Acid Composition in Each of Them in Urine Samples of Rats Fed with a Standard Diet or a High-Fat High-Sucrose Diet

In urine samples, we observed a decrease in total TAG fraction and the level of MUFA in TAG (TAG–CBG: −21.6%, *p* < 0.05, vs. Control group, Table 3; MUFA–CBG: −21.6%, *p* < 0.05, vs. Control group, Table 3). In the HFHS group, we noticed an increase in total TAG content (HFHS: +37.6%, *p* < 0.05, vs. Control group, Table 3). Importantly, CBG administration to rats fed with an HFHS caused a decrease in the level of MUFA, PUFA and total TAG (MUFA–HFHS+CBG: −31.2% and −39.2%, *p* < 0.05, Table 3; PUFA–HFHS+CBG: −35.0% and −33.5%, *p* < 0.05, Table 3; TAG–HFD+CBG: −39.0%, *p* < 0.05, Table 3) than in the Control and/or HFHS group. In DAG fraction, SFA level was reduced in the CBG and HFHS+CBG groups (CBG: −37.3%, HFHS+CBG: −38.3% and −29.2%, *p* < 0.05, Table 3) than in the Control and HFHS groups, respectively. MUFA level in DAG pool was also reduced in all experimental groups (CBG: −59.6%, HFHS: −63.4%, HFHS+CBG: −75.6% and −33.2%, *p* < 0.05, vs. Control and HFHS groups, Table 3). Compared to the Control groups, PUFA content in DAG was decreased (CBG: −51.3%, HFHS: −55.2%, HFHS+CBG: −53.4%, *p* < 0.05, Table 3). Total DAG concentration also declined in all experimental groups (CBG: −41.3%, HFHS: −34.1%, HFHS+CBG: −46.3%, *p* < 0.05, Table 3) in comparison with the Control group. Among FFA fraction, SFA level and total lipid fraction were enhanced in the HFHS group (SFA–HFHS: +64.5%, *p* < 0.05, vs. Control group, Table 3; FFA–HFHS: +57.3%, *p* < 0.05, vs. Control group, Table 3). Similarly, we noticed an enhancement in the level of SFA, MUFA and total FFA fraction (SFA–HFHS+CBG: +50.2%, *p* < 0.05, vs. Control group, Table 3; MUFA–HFHS+CBG: +23.0%, *p* < 0.05, vs. Control group, Table 3; FFA–HFHS+CBG: +45.0%, *p* < 0.05, vs. Control, group, Table 3). In PL pool, SFA and total PL fraction were higher in the HFHS group (SFA–HFHS: +42.4%, *p* < 0.05, Table 3; PL–HFHS: +35.9%, *p* < 0.05, Table 3) in relation to the Control group. Similarly, in the HFHS+CBG group, increases in PL’s SFA, MUFA, PUFA and total PL levels were marked (SFA–HFHS+CBG: +63.4%, *p* < 0.05, Table 3; MUFA–HFHS+CBG: +26.0% and +24.3%, *p* < 0.05, Table 3; PUFA–HFHS+CBG: +92.0% and +38.7%, *p* < 0.05, Table 3; PL–HFHS+CBG: +59.8% and +17.6%, *p* < 0.05, Table 3) in comparison with the Control and HFHS groups, respectively.

In urine samples, in TAG fraction, cannabigerol supplementation to rats fed with a standard rodent chow caused an increase in the content of C24:0, C22:6 (CBG: +42.7%, +89.6%, respectively, *p* < 0.05, vs. Control group, Table S5) and a decrease in the content of C18:1, C18:2 (CBG: −30.5%, −40.0%, respectively, *p* < 0.05, vs. Control group, Table S5). In the same lipid fraction, rats fed with a high-fat high-sucrose diet had a higher level of C14:0, C18:0, C22:6 (HFHS: +65.3%, +152.3%, +56.1%, respectively, *p* < 0.05, vs. Control group, Table S5) and a lower level of C18:2 (HFHS: −26.3%, *p* < 0.05, vs. Control group, Table S5). Moreover, in TAG we observed significant changes in almost every examined FA in the HFHS+CBG group. There were reductions in C14:0, C18:0, C20:0, C22:0, C24:0, C16:1, C18:1, C24:1, C18:2, C18:3, C20:4, C22:6 contents (HFHS+CBG: −36.0%, −37.4%, −32.0% and −38.2%, −41.3%, −39.4%, −33.6% and −37.5%, −24.6% and −31.6%, −46.5% and −52.8%, −43.9% and −23.9%, −38.3% and −36.3%, −28.8% and −37.6%, −68.3% and −79.7%, respectively, *p* < 0.05, Table S5) compared to the Control and/or HFHS groups, respectively. Only C18:0 concentration in TAG was enhanced in the HFHS+CBG group (HFHS+CBG: +57.9%, *p* < 0.05, Table S5) in relation to the HFHS group.

In urine samples of rats subjected to a CBG only, we noticed a decline in the content of C14:0, C16:0, C16:1, C18:1, C18:2 in DAG (CBG: −37.9%, −31.9%, −48.7%, −67.4%, −58.5%, respectively, *p* < 0.05, vs. Control group, Table S6). Similarly, DAG’s C16:1, C18:1, C18:2, C18:3 levels were decreased in the HFHS group (HFHS: −58.2%, −70.9%, −65.1%, −43.8%, respectively, *p* < 0.05, vs. Control group, Table S6). Compared to the Control and/or HFHS groups, in the HFHS+CBG group, DAG’s content of C14:0, C16:0, C20:0, C24:0, C16:1, C18:1, C18:2, C18:3 was reduced (HFHS+CBG: −33.9%, −36.7% and −35.3%, −53.0% and −46.0%, −38.8% and −27.0%, −63.0%, −81.0% and −34.6%, −72.8%, −51.3%, respectively, *p* < 0.05, Table S6). In the HFHS+CBG group, an enhancement in C20:4 content in DAG was observed (HFHS+CBG: +136.2% and +65.7%, *p* < 0.05, vs. Control and HFHS groups, respectively, Table S6).

In FFA fraction, CBG supplementation only caused an increase in C20:0 level (CBG: +23.8%, *p* < 0.05, vs. Control group, Table S7) and decreases in C16:0, C18:2 levels (CBG: −27.5%, −37.3%, respectively, *p* < 0.05, vs. Control group, Table S7). In FFA pool, rats from the HFHS group had a higher level of C16:0, C18:0, C24:0, C18:1 (HFHS: +65.3%, +80.6%, +46.3%, +34.2%, respectively, *p* < 0.05, vs. Control group, Table S7) and a lower level of C16:1 (HFHS: −31.8%, *p* < 0.05, vs. Control group, Table S7). In the HFHS+CBG group, an increment in FFA’s C16:0, C18:0, C20:0, C18:1 concentrations (HFHS+CBG: +49.7%, +61.9%, +28.6%, +36.6%, respectively, *p* < 0.05, Table S7) and a decline in FFA’s C16:1 concentration (HFHS+CBG: −44.0%, *p* < 0.05, Table S7) were observed in relation to the Control group.

Lastly, in urine samples, PL fraction disclosed an enhancement in the content of C18:0, C20:0, C22:0, C24:0, C16:1, C18:3, C22:6 in the HFHS group (HFHS: +54.9%, +37.0%, +36.8%, +22.1%, +41.8%, +34.6%, +20.2%, respectively, *p* < 0.05, vs. Control group, Table S8). Moreover, C16:0, C18:0, C16:1, C18:1, C18:2, C20:4, C22:6 levels in PL were greater in rats fed with an HFHS and CBG (HFHS+CBG: +42.5%, +92.0%, +31.5%, +35.2% and +41.2%, +27.4%, +126.6% and +58.8%, +29.4%, respectively, *p* < 0.05, Table S8) than in the Control and/or HFHS groups, respectively. In the same experimental group, the content of C20:5 in PL was diminished (HFHS+CBG: −30.1%, *p* < 0.05, vs. Control group, Table S8).

### 3.3. Cannabigerol Influence on the Expression of Fatty Acid Transporters in the Kidney Tissue of Rats Fed with a Standard Diet or a High-Fat High-Sucrose Diet

**Figure 1:**
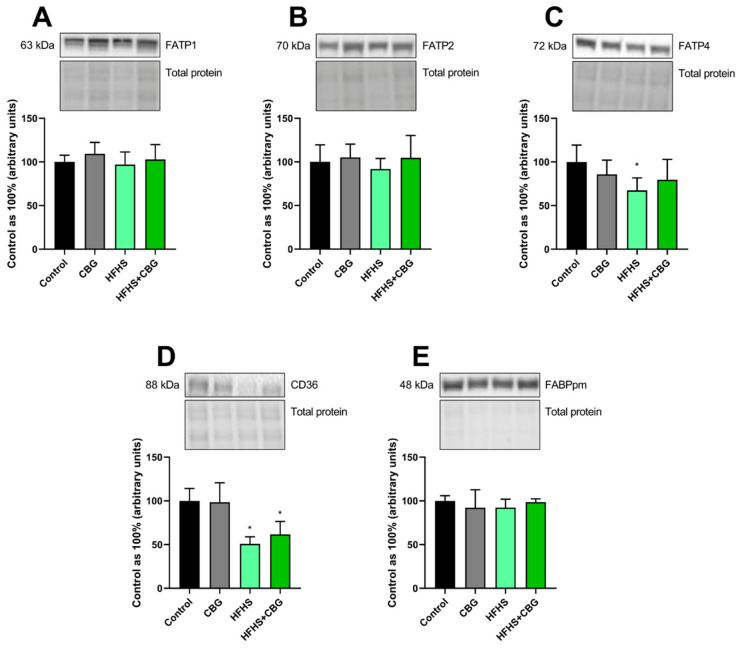
Cannabigerol (CBG) influence on the expression of fatty acid transporter 1 (FATP1, (**A**)), fatty acid transporter 2 (FATP2, (**B**)), fatty acid transporter 4 (FATP4, (**C**)), fatty acid translocase (CD36, (**D**)) and plasma membrane fatty acid-binding protein (FABPpm, (**E**)) in the kidney tissue of rats fed with a standard diet (Control) or a high-fat high-sucrose diet (HFHS). * *p* < 0.05—significant difference between CBG, HFHS and HFHS+CBG groups vs. Control group; *p* < 0.05—significant difference between HFHS+CBG group vs. HFHS group.

In the kidney tissue, a high-fat high-sucrose feeding caused a decrease in the expression of fatty acid transporter 4 (FATP4) and fatty acid translocase (CD36) (FATP4–HFHS: −32.5%, *p* < 0.05, vs. Control group, Figure 1C; CD36–HFHS: −56.2%, *p* < 0.05, vs. control group, Figure 1D). In the HFHS+CBG group, CD36 expression was lower (HFHS+CBG: −46.9%, *p* < 0.05, Figure 1D) than in the Control group. The expression of fatty acid transporters 1 and 2 (FATP1 and FATP2) and plasma membrane fatty acid-binding protein (FABPpm) remained significantly unchanged (*p >* 0.05, Figure 1A,B,E).

### 3.4. Cannabigerol Influence on the Expression of Proteins Regulating Fatty Acid Metabolism in the Kidney Tissue of Rats Fed with a Standard Diet or a High-Fat High-Sucrose Diet

**Figure 2:**
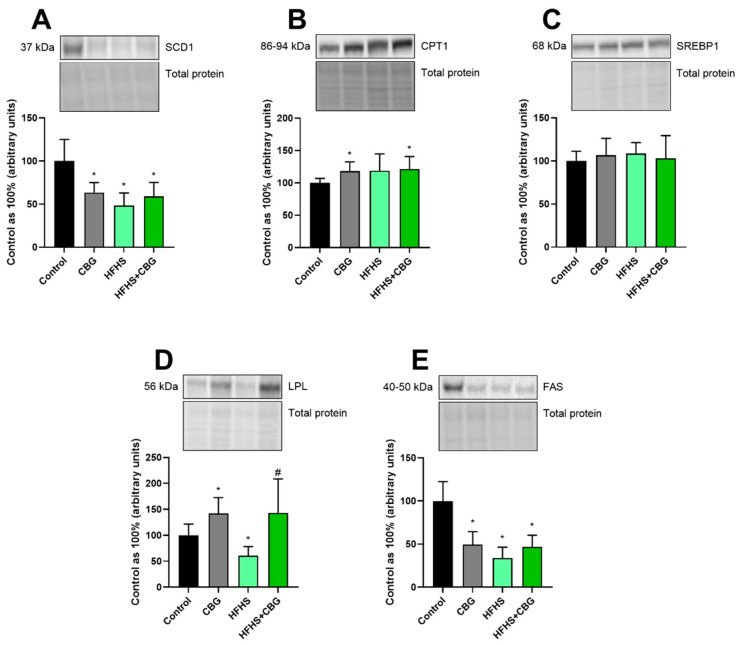
Cannabigerol (CBG) influence on the expression of stearoyl-CoA desaturase 1 (SCD1, (**A**)), carnitine palmitoyltransferase 1 (CPT1, (**B**)), sterol regulatory element-binding transcription factor 1 (SREBP1, (**C**)), lipoprotein lipase (LPL, (**D**)) and fatty acid synthase (FAS, (**E**)) in the kidney tissue of rats fed with a standard diet (Control) or a high-fat high-sucrose diet (HFHS). * *p* < 0.05—significant difference between CBG, HFHS and HFHS+CBG groups vs. Control group; # *p* < 0.05—significant difference between HFHS+CBG group vs. HFHS group.

In the kidney tissue, the expression of stearoyl-CoA desaturase 1 (SCD1) and fatty acid synthase (FAS) was declined in all experimental groups (SCD1–CBG: −36.5%, HFHS: −51.5%, HFHS+CBG: −40.8%, *p* < 0.05, vs. Control group, Figure 2A; FAS–CBG: −50.5%, HFHS: −66.1%, HFHS+CBG: −53.3%, *p* < 0.05, vs. Control group, Figure 2E). Differently, an enhancement in carnitine palmitoyltransferase 1 (CPT1) expression was noticed in rats treated with CBG (CBG: +18.2%, HFHS+CBG: +21.2%, *p* < 0.05, vs. Control group, Figure 2B). In addition, sterol regulatory element-binding transcription factor 1 (SREBP1) expression remained unchanged (*p* > 0.05, Figure 2C). Lastly, the expression of lipoprotein lipase (LPL) was lower in rats from the HFHS group (HFHS: −39.3%, *p* < 0.05, vs. Control group, Figure 2D), while cannabigerol supplementation restored LPL expression (CBG: +42.4%, HFHS+CBG: +135.7%, *p* < 0.05, vs. Control or HFHS groups, respectively, Figure 2D).

### 3.5. Cannabigerol Influence on the Elongation Ratios of Fatty Acids in the Kidney Tissue of Rats Fed with a Standard Diet or a High-Fat High-Sucrose Diet

**Figure 3:**
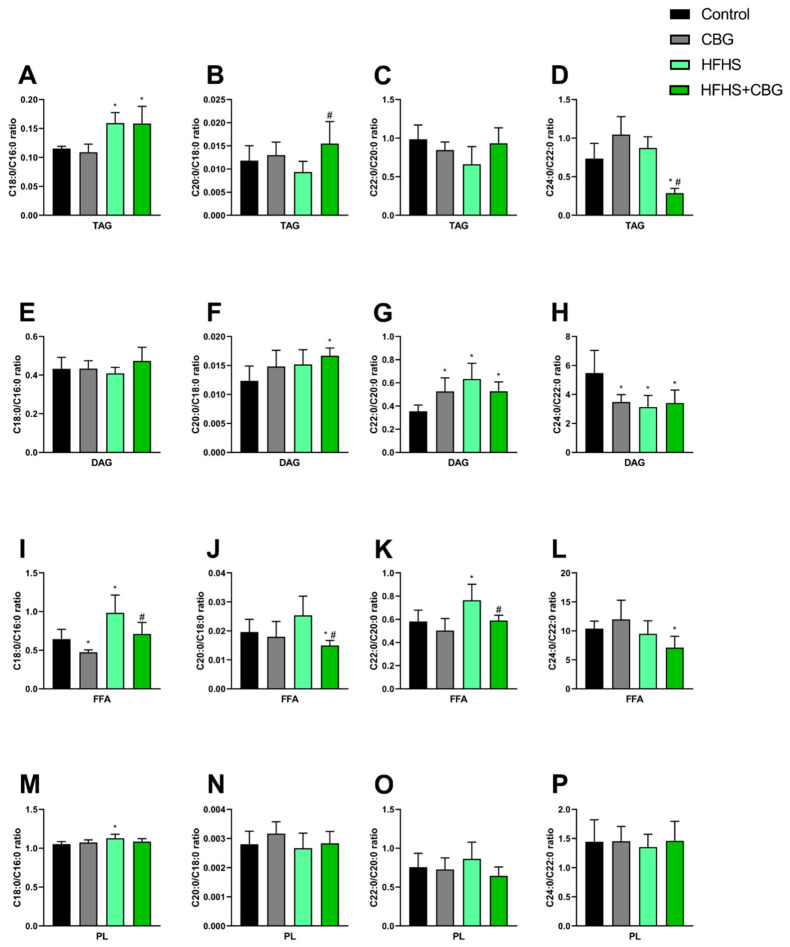
Cannabigerol (CBG) influence on the elongation ratios of fatty acids as follows: C18:0/C16:0 (**A**,**E**,**I**,**M**), C20:0/C18:0 (**B**,**F**,**J**,**N**), C22:0/C20:0 (**C**,**G**,**K**,**O**), C24:0/C22:0 (**D**,**H**,**L**,**P**) in triacylglycerol (TAG), diacylglycerol (DAG), free fatty acid (FFA) and phospholipid (PL) in the kidney tissue of rats fed with a standard diet (Control) or a high-fat high-sucrose diet (HFHS). * *p* < 0.05—significant difference between CBG, HFHS and HFHS+CBG groups vs. Control group; # *p* < 0.05—significant difference between HFHS+CBG group vs. HFHS group.

In the kidney tissue, the C18:0/C16:0 ratio in TAG was enhanced in rats that received an HFHS diet (HFHS: +38.4%, HFHS+CBG: +37.8%, *p* < 0.05, vs. Control group, Figure 3A). In TAG, the ratio of C20:0/C18:0 was also increased in the HFHS+CBG group (HFHS+CBG: +63.7%, *p* < 0.05, vs. HFHS group, Figure 3B). In the same experimental group, the C24:0/C22:0 ratio in TAG was decreased (HFHS+CBG: −60.9% and −67.1%, *p* < 0.05, Figure 3D) compared to the Control and HFHS groups, respectively. DAG’s C20:0/C18:0 ratio was increased after HFHS with CBG supplementation (HFHS+CBG: +33.8%, *p* < 0.05, vs. Control group, Figure 3F). In all experimental subjects, the C22:0/C20:0 ratio in DAG was higher (CBG: +48.4%, HFHS: +79.0%, HFHS+CBG: +48.8%, *p* < 0.05, Figure 3G) than in the Control group. Differently, a decrease in the C24:0/C22:0 ratio in DAG was observed in all examined groups (CBG: −36.5%, HFHS: −43.0%, HFHS+CBG: −37.9%, *p* < 0.05, vs. Control group, Figure 3H). In FFA fraction, the C18:0/C16:0 ratio was higher in rats from the HFHS group (HFHS: +52.7%, *p* < 0.05, vs. Control group, Figure 3I), while CBG supplementation attenuated the C18:0/C16:0 value (CBG: −26.5%, HFHS: −27.6%, *p* < 0.05, vs. Control or HFHS groups, respectively, Figure 3I). Rats fed with an HFHS diet with the addition of CBG had a lower C20:0/C18:0 ratio in FFA (HFHS+CBG: −22.5% and −40.8%, *p* < 0.05, vs. Control and HFHS groups, respectively, Figure 3J). FFA’s C20:0/C18:0 ratio was enhanced in HFHS-fed rats (HFHS: +31.5%, *p* < 0.05, vs. Control group, Figure 3K) while CBG supplementation attenuated its value (HFHS+CBG: −22.8%, *p* < 0.05, vs. Control group, Figure 3K). In the HFHS+CBG group, the ratio of C24:0/C22:0 in FFA was reduced (HFHS+CBG: −31.6%, *p* < 0.05, Figure 3L) in relation to the Control group. In PL fraction, the C18:0/C16:0 ratio was increased after administration of HFHS (HFHS: +7.2%, *p* < 0.05, vs. Control group, Figure 3M).

### 3.6. Cannabigerol Influence on the Desaturation Ratios of Fatty Acids in the Kidney Tissue of Rats Fed with a Standard Diet or a High-Fat High-Sucrose Diet

**Figure 4:**
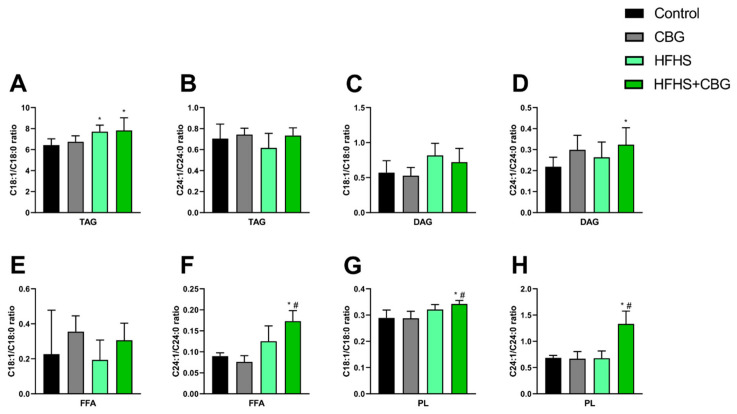
Cannabigerol (CBG) influence on the desaturation ratios of fatty acids as follows: C18:1/C18:0 (**A**,**C**,**E**,**G**), C24:1/C24:0 (**B**,**D**,**F**,**H**) in triacylglycerol (TAG), diacylglycerol (DAG), free fatty acid (FFA) and phospholipid (PL) in the kidney tissue of rats fed with a standard diet (Control) or a high-fat high-sucrose diet (HFHS). * *p* < 0.05—significant difference between CBG, HFHS and HFHS+CBG groups vs. Control group; # *p* < 0.05—significant difference between HFHS+CBG group vs. HFHS group.

In TAG pool, the C18:1/C18:0 ratio was increased in rats fed with an HFHS (HFHS: +19.6%, HFHS+CBG: +21.5%, *p* < 0.05, vs. Control group, Figure 4A). TAG’s C24:1/C24:0 ratio and DAG’s C18:1/C18:0 ratio remained statistically unchanged (*p >* 0.05, Figure 4B,C). In the HFHS+CBG group, the C24:1/C24:0 ratio in DAG fraction was higher (HFHS+CBG: +48.4%, *p* < 0.05, Figure 4D) than in the Control group. C18:1/C18:0 value in FFA did not statistically change (*p >* 0.05, Figure 4E). In the HFHS+CBG group, the C24:1/C24:0 value in FFA was increased (HFHS+CBG: +93.4% and +38.3%, *p* < 0.05, Figure 4F) in comparison to the Control and HFHS groups, respectively. PL’s C18:1/C18:0 value was enhanced by the HFHS diet with the addition of CBG (HFHS+CBG: +18.4% and +6.5%, *p* < 0.05, vs. Control and HFHS groups, respectively, Figure 4G). In PL fraction, we also observed an increase in the value of C24:1/C24:0 (HFHS+CBG: +94.1% and +96.4%, *p* < 0.05, Figure 4H) in comparison with the Control and HFHS groups, respectively.

### 3.7. Cannabigerol Influence on the De Novo Lipogenesis Ratio of Fatty Acids in the Kidney Tissue of Rats Fed with a Standard Diet or a High-Fat High-Sucrose Diet

**Figure 5:**
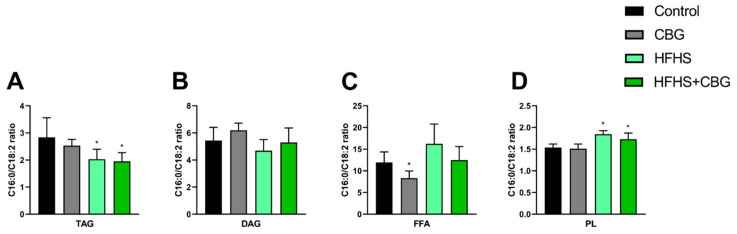
Cannabigerol (CBG) influence on the de novo lipogenesis ratio of C16:0/C18:2 in triacylglycerol (TAG) (**A**), diacylglycerol (DAG)) (**B**), free fatty acid (FFA)) (**C**) and phospholipid (PL)) (**D**) in the kidney tissue of rats fed with a standard diet (Control) or a high-fat high-sucrose diet (HFHS). * *p* < 0.05—significant difference between CBG, HFHS and HFHS+CBG groups vs. Control group; # *p* < 0.05—significant difference between HFHS+CBG group vs. HFHS group.

C16:0/C18:2 index in TAG declined in rats subjected to an HFHS diet (HFHS: −28.4%, HFHS+CBG: −31.3%, *p* < 0.05, vs. Control group, Figure 5A). CBG supplementation also reduced the de novo lipogenesis index of C16:0/C18:2 in FFA (CBG: −30.1%, *p* < 0.05, vs. Control group, Figure 5C). However, C16:0/C18:2 index in PL was enhanced under high-fat high-sucrose feeding (HFHS: +20.1%, HFHS+CBG: +12.6%, *p* < 0.05, vs. Control group, Figure 5A).

### 3.8. Cannabigerol Influence on the Histological Results of the Kidney Tissue of Rats Fed with a Standard Diet or a High-Fat High-Sucrose Diet

**Figure 6:**
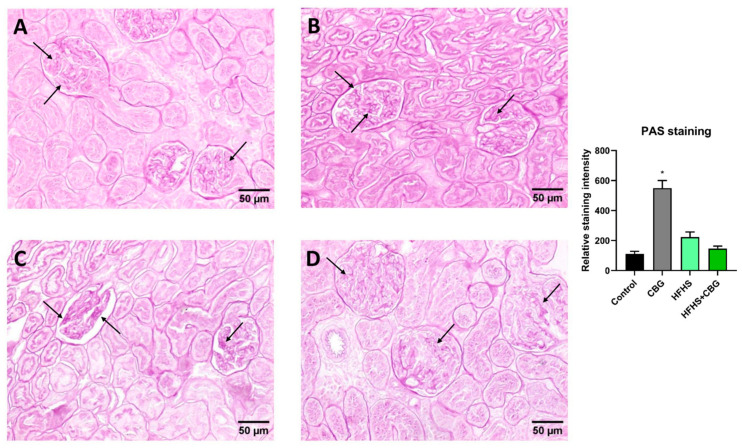
Periodic Acid-Schiff staining (PAS) analysis of the kidney tissue of rats fed with a standard diet (Control, (**A**)), cannabigerol (CBG, (**B**)), high-fat high-sucrose diet (HFHS, (**C**)) and high-fat high-sucrose diet with cannabigerol (HFHS+CBG, (**D**)) and PAS staining intensity. Arrows show the morphological changes. * *p* < 0.05—significant difference between CBG, HFHS and HFHS+CBG groups vs. Control group; # *p* < 0.05—significant difference between HFHS+CBG group vs. HFHS group.

In the CBG group, the basement membranes of the glomeruli and tubules were distinctly separated (Figure 6B). Importantly, in the HFHS group, PAS staining revealed changes in the structure of the kidneys, indicating an enlargement of the mesangial matrix and/or a thickening of the glomerular basement membrane (Figure 6C). Rats that received an experimental diet, along with CBG, demonstrated a reduction in changes in the glomeruli and tubules morphology than rats in the HFHS diet group, indicating better preservation of the basement membrane structure (Figure 6D).

**Figure 7:**
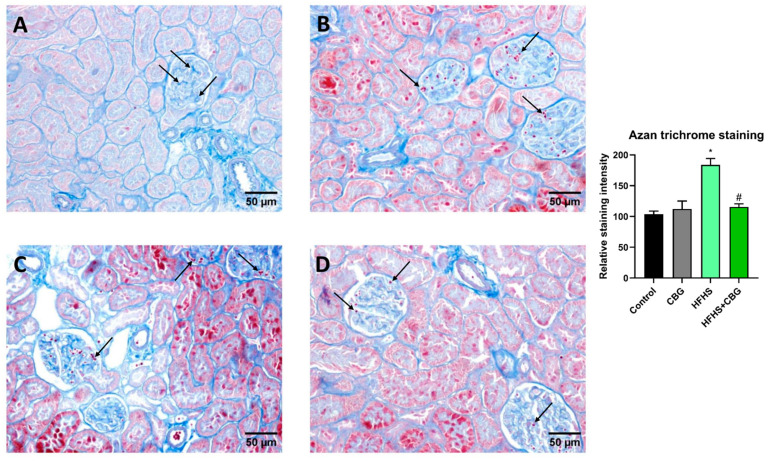
AZAN trichrome staining of the kidney tissue of rats fed with a standard diet (Control, (**A**)), cannabigerol (CBG, (**B**)), high-fat high-sucrose diet (HFHS, (**C**)) and high-fat high-sucrose diet with cannabigerol (HFHS+CBG, (**D**)) and azan trichrome staining intensity. Arrows show the morphological changes. * *p* < 0.05—significant difference between CBG, HFHS and HFHS+CBG groups vs. Control group; # *p* < 0.05—significant difference between HFHS+CBG group vs. HFHS group.

Azan trichrome staining of the kidney from the group treated with CBG showed a similar renal structure to that in the Control group. Minimal collagen deposition in the interstitial spaces was noted. There were no indications of tubulointerstitial fibrosis (Figure 7B). In the HFHS group, kidney slides revealed significant histopathological changes. There was an increased presence of azan-positive collagen fibers in the interstitial regions, which is indicative of tubulointerstitial fibrosis. Some glomeruli also showed structural irregularities, such as slight mesangial expansion and initial signs of glomerulosclerosis (Figure 7C). In rats that received the experimental diet along with CBG treatment, renal structure was somewhat maintained compared to the HFHS group. A significant decrease in the accumulation of interstitial collagen was noted, suggesting a reduction in tubulointerstitial fibrosis. Glomerular changes were also less prominent with reduced signs of mesangial expansion and glomerulosclerosis (Figure 7D).

### 3.9. Cannabigerol Influence on the Functional Renal Markers in Urine Samples of Rats Fed with a Standard Diet or a High-Fat High-Sucrose Diet

**Figure 8:**
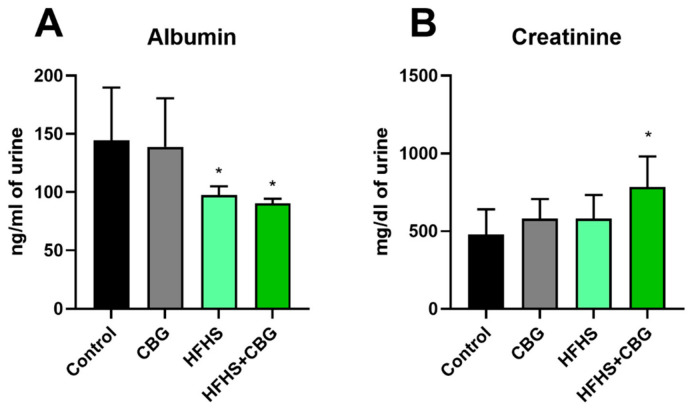
Cannabigerol (CBG) influence on the concentration of functional renal markers: albumin (**A**) and creatinine (**B**) in urine samples of rats fed with a standard diet (Control) or a high-fat high-sucrose diet (HFHS). * *p* < 0.05—significant difference between CBG, HFHS and HFHS+CBG groups vs. Control group; # *p* < 0.05—significant difference between HFHS+CBG group vs. HFHS group.

In the urine sample, the concentration of albumin was lower in rats fed with an experimental diet alone or in the addition of cannabigerol (HFHS: −32.5%, HFHS+CBG: −37.5%, *p* < 0.05, Figure 8A) than in the Control group. However, in the HFHS+CBG group, urinary concentration of creatinine was higher (HFHS+CBG: +63.7%, *p* < 0.05, Figure 8B) than in the Control group.

### 3.10. Cannabigerol Influence on the Expression of Selected Proteins Involved in Lipid Metabolism and Antioxidant Defense in the Kidney Tissue of Rats Fed with a Standard Diet or a High-Fat High-Sucrose Diet

**Figure 9:**
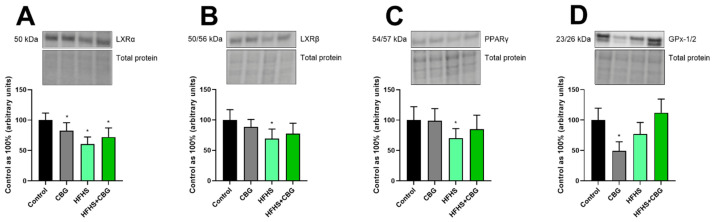
Cannabigerol (CBG) influence on the expression of selected proteins involved in lipid metabolism and antioxidant defense: liver X receptor alpha (LXRα, (**A**)), liver X receptor beta (LXRβ, (**B**)), peroxisome proliferator-activated receptor gamma (PPARγ, (**C**)) and glutathione peroxidase (GPx-1/2, (**D**)) in the kidney tissue of rats fed with a standard diet (Control) or a high-fat high-sucrose diet (HFHS). * *p* < 0.05—significant difference between CBG, HFHS and HFHS+CBG groups vs. Control group; # *p* < 0.05—significant difference between HFHS+CBG group vs. HFHS group.

In the kidney tissue, the expression of liver X receptor alpha (LXRα) was declined in all experimental groups (CBG: −17.6%, HFHS: −39.4%, HFHS+CBG: −28.0%, *p* < 0.05, vs. Control group, Figure 9A). Moreover, a reduction in liver X receptor beta (LXRβ) expression and peroxisome proliferator-activated receptor gamma (PPARγ) was noticed in rats fed with an experimental diet only (LXRβ–HFHS: −30.8%, *p* < 0.05, vs. Control group, Figure 9B; PPARγ–HFHS: −29.9%, *p* < 0.05, vs. Control group, Figure 9C). Differently, cannabigerol supplementation to rats fed with a standard rodent chow decreased glutathione peroxidase (GPx-1/2) expression (CBG: −50.6%, *p* < 0.05, vs. Control group, Figure 9D).

## 4. Discussion

An HFHS diet induces metabolic disturbances, which lead to structural and biochemical changes in the kidney. PAS staining is used to demonstrate carbohydrate-rich pathological changes such as glomerular basement membrane thickening, mesangial expansion, and glycogen accumulation. A diet rich in fat alone increases lipid accumulation in the kidneys, while coexistent sucrose excess intake may expedite metabolic alterations [26,27]. In addition to exposure of the kidneys to fats in the blood, they are subjected to elevated lipid contents in urine, which are reabsorbed. Those changes result in modifications in the metabolic state of the kidneys and also disrupt lipid fraction composition, affecting lipid oxidation, therefore triggering oxidative stress [12,28]. In our study, histological AZAN staining revealed collagen accumulation within the mesangium and capillary tufts due to prolonged metabolic injury. Renal metabolism of lipids is possible by cooperation between several transporters, enzymes and other proteins. FA uptake is determined by FA-transport proteins (FATP), plasma membrane FA-binding protein (FABPpm) and FA translocase (CD36). Within the first group, the most prominent in physiological conditions in proximal tubules is FATP2, with FATP1 and FATP4 expressed in modest amounts [29]. Our study did not reveal any differences in FATP1 and FATP2 expressions in any experimental group; however, compared to the control group, a significant decrease in FATP4 expression was observed in rodents fed with an HFHS diet. Fatty acid oxidation (FAO) in early kidney disorders is impaired; therefore less acyl-CoA is transformed into acetyl-CoA in order to incorporate it into the citric acid cycle and may be linked with a significant decrease in FATP4 activity and expression [30,31]. Under CBG administration to rats fed with an HFHS diet, the expression of FATP4 was not substantially changed. FATP4 expression is extensively connected to peroxisome proliferator activated receptors (PPAR) pathways [32]. The following carrier, which levels are positively correlated with tubular injury and the course of fibrosis, is CD36 [33]. Overexpression of CD36 alone is thought to be insufficient to generate renal fibrosis, it is definitely an important contributing factor [34]. Contrary to what previous studies have shown, in the herein study, CD36 expression was significantly decreased in rats subjected to an HFHS diet [35,36]. To further elucidate this finding, we additionally assessed upstream transcriptional regulators of CD36 and found that PPARγ and both liver X receptors LXRα and LXRβ were significantly downregulated in the HFHS group, consistent with suppressed CD36 expression and suggesting reduced activation of nuclear receptor-dependent lipid-sensing pathways. Among these, in which substantial alterations occurred, MUFA level in FFA and PL stand out particularly in the context of our experiment. A great decrease relative to the Control group in the first of the two discussed fractions (FFA, PL) was recorded in the experimental groups. These MUFA in FFA can be incorporated into TAG to be stored, as this form is preferred for storage and coincidentally functions as a protective mechanism in the case of excess lipid supply [37,38]. There was also an increase in C18:1/C18:0 desaturation ratio in TAG, in both groups receiving HFHS diet (HFHS, HFHS+CBG), which could further support these assumptions. Additionally, a simultaneous decrease in stearoyl-CoA desaturase (SCD1) expression in the mentioned groups could indicate diminished transformation of SFA into MUFA [39]. Moreover, what was observed in FFA in the CBG and HFHS+CBG groups, but did not appear in the case of HFHS, was an increase in PUFA. Serving as precursors of various lipid mediators, PUFA in FFA are crucial for preserving renal perfusion and modulation of inflammatory pathways and fibrosis, which could be related to why such shifts in MUFA occurred in both groups under CBG supplementation [40]. In the case of phospholipid, an increase in MUFA level in the HFHS+CBG is consistent with C18:1/C18:0 and C24:1/C24:0 desaturation ratios. Within the same experimental group, PUFA level in PL was higher compared to the HFHS group, in which PUFA content was significantly decreased compared to the Control group. These changes may also be related to the aforementioned fact that one of PUFA functions performed is being the source of arachidonic acid (AA; C20:4), subsequently converted into prostaglandins, which regulate the kidneys’ proper activity [41]. In our previous publication, we reported AA content under CBG supplementation to obese rats. We noticed a significant decrease in AA concentration with simultaneous enhancement in 12/15-lipoxygenase expression (expression responsible for the synthesis of anti-inflammatory lipoxin series 4), suggesting that CBG may have potential anti-inflammatory effects [42]. What is more, in the HFHS group in urine samples, the levels of SFA, MUFA and PUFA were independently elevated in every fraction (except for DAG) compared to the Control group. These modifications transpiring in the HFHS group were different from those observed with CBG, which indicates its inhibiting and potential protective functions against the development of renal disorder. In patients with diabetic kidney disease, disturbances in both glomerular filtration and tubular reabsorption are observed. This results in augmented urinary excretion of proteins, glucose and lipids [43]. A decrease in urinary TAG level was observed in rats treated with an HFHS and CBG compared to HFHS alone. Similar changes were also observed in DAG levels, but the opposite was true for PL, where urinary excretion increased with HFHS and CBG compared to HFHS alone. This lipiduria, most commonly seen in nephrotic syndrome, is clearly correlated with filtration membrane damage, so decreases in TAG and DAG levels after CBG treatment could indicate improved filtration membrane function [44].

Lipid metabolism is also reliant on several enzymes, allowing renal cells to utilize lipids in several pathways, including FA storage and oxidation, required to maintain proper kidney activity. One of the most frequently referred to is carnitine palmitoyltransferase 1 (CPT1), as it is considered to be rate-limiting. A study conducted by D’Aniello et al. stated that similarly to fenofibrate, cannabigerol alone cannot up-regulate PPARα [45]; however, acting as its agonist, it can induce the expression of lipid metabolism pathway enzymes, e.g., CPT1, which was observed in the herein study [46]. We have also observed a decrease in amounts of fatty acid synthase (FAS)—an enzyme catalyzing the process of de novo FA synthesis [47]. Changes related to de novo lipogenesis (DNL) can be seen in the index of C16:0/C18:2, where palmitic acid is a major product of FA synthesis [48]. Following the reduction in FAS expression, a concomitant decrease in the DNL index was observed in both the HFHS and HFHS+CBG groups in TAG, as well as CBG in FFA. We suppose that CBG, through its reliable antioxidant properties, may protect UFA against adverse oxidation, similarly to other cannabinoids [49,50,51,52,53]. In our study, cannabigerol has also demonstrated activity with an impact on the extent of the enzyme responsible for lipolysis—lipoprotein lipase (LPL), which is opposite to what is observed in the subjects undergoing the HFHS diet alone. Previous studies provide evidence for a negative relation of decreased LPL level in kidney tissue in patients with DN to deterioration of renal function and inflammatory response, including markers like TNF-α and activation and aggregation of pro-inflammatory M1 macrophages [54,55]. We observed that the expression of lipase responsible for the hydrolysis of TAG to FFA and glycerol was significantly elevated with the co-administration of CBG. This dietary addition lowered the total amount of TAG in renal cells, simultaneously raising total FFA. It remains unclear whether the observed changes in lipid composition result directly from increased LPL activity or from other CBG-mediated metabolic adaptations. Nevertheless, the concomitant reduction in lipid accumulation and alterations in fatty acid profiles suggest that CBG may influence renal lipid handling under HFHS conditions [56,57]. A decrease in the amount of MUFA in FFA was observed in all experimental groups compared to the control group, while a significant increase in PL’s MUFA level in the HFHS+CBG group compared to both the Control and HFHS groups was reported. Mechanisms through which MUFA attenuate damage induced by SFA are not completely acknowledged; however they can enhance the integration of FA into neutral lipids, thus reducing the amount of SFA and consecutive toxic lipid metabolites, such as DAG [58]. This is executed through faster incorporation of MUFA compared to SFA into TAG, resulting in mitigation of cell stress response as a consequence of lipid oversupply [59]. These desaturation reactions are executed by SCD1, which decrease was recorded in each group and relatively more pronounced in the HFHS group only. Interestingly, the decrease in SCD1 expression observed in the CBG, HFHS, and HFHS+CBG groups was not accompanied by significant changes in SREBP1 expression, suggesting that the regulation of SCD1 may occur independently of changes in SREBP1 abundance and may be a result of multiple nutritional, hormonal and metabolic signals.

Interestingly, urinary albumin levels were significantly lower in both HFHS-fed groups compared to the Control group. This finding was unexpected, as increased urinary albumin excretion is commonly associated with progressive renal injury. However, it should be noted that in the present study HFHS model represents an early stage of metabolic kidney injury rather than advanced renal disease characterized by albuminuria. Therefore, urinary albumin did not parallel the molecular, lipidomic and histological alterations observed in renal tissue. This discrepancy is a limitation of the study and needs to be further investigated in an animal model with longer experimental feeding to induce diabetic kidney disease with more advanced renal pathology.

A limitation of the present study is that only male rats were included. This approach was chosen to minimize variability associated with the estrous cycle, which can influence metabolic and inflammatory responses. Another limitation of the present study is the incomplete evaluation of renal function in healthy animals receiving CBG. Owing to limited sample availability, serum renal function markers were not assessed. Future studies should include both serum and urinary markers to provide a more comprehensive evaluation of the renal safety profile of CBG and sex-specific profile in renal lipid metabolism.

## 5. Conclusions

Finally, we managed to highlight several changes between the HFHS and HFHS+CBG groups, which display the differences in lipid profile that arise when CBG is supplemented with a diet associated with Western populations [60]. Among the most notable were changes in the lipid composition of the kidney, where a significant increase was observed in three instances: PUFA in FFA and MUFA, PUFA in PL. Dissimilar changes, however, occurred in urinary lipids, as a decrease was observed in SFA and MUFA in DAG, and MUFA, PUFA in TAG and the total fraction. Furthermore, the exact opposite changes appeared in PL, where MUFA, PUFA and also the total amount have increased. Although the lipidome did display alterations in several cases, there were no changes observed between those groups in the expression of transporters involved in lipid absorption into the cell, but a significant increase was observed in one of the enzymes involved in lipid metabolism—LPL expression. Despite not observing an increase in SCD1 expression, both phospholipid desaturation ratios and the C24:1/C24:0 ratio in FFA increased, indicating an increased desaturation of selected FA in these lipid fractions. The findings of the presented study demonstrate a significant effect of CBG on lipid metabolism in the kidneys. The modulation of lipid metabolic pathways, together with alterations in inflammatory and fibrosis markers (previously published [42]), suggests that CBG can be involved in kidney disorders.

## Figures and Tables

**Table 2:** Cannabigerol (CBG) influence on the content of saturated fatty acids (SFA), monounsaturated fatty acids (MUFA) and polyunsaturated fatty acids (PUFA) in triacylglycerol (TAG), diacylglycerol (DAG), free fatty acid (FFA) and phospholipid (PL) fractions and total content of mentioned lipid fractions in the kidney tissue of rats subjected to a standard diet (Control) or a high-fat high-sucrose diet (HFHS).

		Control	CBG	HFHS	HFHS+CBG
TAG	SFA	52,234.8 ± 14,391.9	23,055.0 ± 6854.5 *	52,434.8 ± 7013.6	46,811.4 ± 11,068.4
	MUFA	47,443.4 ± 11,697.1	34,252.8 ± 10,650.2	57,079.0 ± 5368.2	57,791.3 ± 13,773.8
	PUFA	12,697.7 ± 3495.3	12,588.0 ± 3440.5	18,427.9 ± 5452.9	14,963.3 ± 4556.5
	Total	137,937.3 ± 19,158.5	46,511.6 ± 10,524.7 *	137,736.3 ± 24,395.4	127,296.5 ± 33,935.3
DAG	SFA	492.2 ± 20.8	472.5 ± 40.3	520.3 ± 59.3	473.0 ± 37.1
	MUFA	132.3 ± 21.2	96.1 ± 19.3 *	142.0 ± 37.0	123.9 ± 30.8
	PUFA	175.7 ± 23.4	135.8 ± 7.3 *	168.2 ± 33.0	162.2 ± 15.0
	Total	795.9 ± 84.9	704.4 ± 53.3	747.7 ± 98.3	744.6 ± 96.3
FFA	SFA	349.0 ± 69.6	357.1 ± 22.5	429.6 ± 74.3	421.0 ± 38.6
	MUFA	117.2 ± 24.4	60.6 ± 15.2 *	55.0 ± 12.4 *	72.1 ± 19.6 *
	PUFA	31.7 ± 7.1	75.1 ± 14.0 *	30.8 ± 4.4	51.7 ± 13.6 ^#^
	Total	444.9 ± 106.8	492.3 ± 44.9	550.9 ± 138.0	587.2 ± 78.4
PL	SFA	23,650.8 ± 1504.3	23,803.8 ± 1666.3	22,981.1 ± 1310.3	23,962.6 ± 1369.3
	MUFA	4041.1 ± 391.9	4042.3 ± 231.7	4239.3 ± 168.7	4582.8 ± 300.4 * ^#^
	PUFA	26,686.8 ± 2575.9	27,653.6 ± 2186.8	22,895.4 ± 2230.0 *	27,023.4 ± 1987.9 ^#^
	Total	54,378.6 ± 4127.7	55,499.7 ± 3877.6	50,115.7 ± 3289.7	54,057.5 ± 5511.4

The values are expressed in nanomoles per gram of tissue. * *p* < 0.05—significant difference between CBG, HFHS and HFHS+CBG groups vs. Control group; # *p* < 0.05—significant difference between HFHS+CBG group vs. HFHS group.

**Table 3:** Cannabigerol (CBG) influence on the content of saturated fatty acids (SFA), monounsaturated fatty acids (MUFA) and polyunsaturated fatty acids (PUFA) in triacylglycerol (TAG), diacylglycerol (DAG), free fatty acid (FFA) and phospholipid (PL) fractions and total content of mentioned lipid fractions in urine samples of rats subjected to a standard diet (Control) or a high-fat high-sucrose diet (HFHS).

		Control	CBG	HFHS	HFHS+CBG
TAG	SFA	24.7 ± 5.0	22.1 ± 2.9	33.7 ± 10.7	26.4 ± 4.6
	MUFA	36.4 ± 5.3	26.6 ± 5.3 *	42.3 ± 11.3	25.9 ± 4.6 * ^#^
	PUFA	14.0 ± 2.8	11.0 ± 1.9	13.7 ± 3.8	9.1 ± 2.0 * ^#^
	Total	76.3 ± 12.9	59.8 ± 10.1 *	105.0 ± 20.6 *	64.0 ± 13.6 ^#^
DAG	SFA	36.7 ± 10.6	23.0 ± 3.7 *	32.0 ± 6.1	22.6 ± 4.4 * ^#^
	MUFA	13.5 ± 2.5	5.4 ± 1.5 *	4.9 ± 1.1 *	3.3 ± 0.7 * ^#^
	PUFA	4.6 ± 0.7	2.3 ± 0.4 *	2.1 ± 0.4 *	2.2 ± 0.6 *
	Total	55.7 ± 14.1	32.7 ± 8.1 *	36.7 ± 8.2 *	30.0 ± 5.4 *
FFA	SFA	14.9 ± 3.0	11.2 ± 2.3	24.6 ± 1.0 *	22.4 ± 3.0 *
	MUFA	1.9 ± 0.3	1.5 ± 0.5	2.3 ± 0.4	2.3 ± 0.3 *
	PUFA	0.9 ± 0.2	0.7 ± 0.1	0.8 ± 0.0	0.8 ± 0.1
	Total	17.6 ± 3.3	13.5 ± 2.7	27.7 ± 1.3 *	25.5 ± 2.8 *
PL	SFA	32.3 ± 4.4	28.2 ± 3.9	46.0 ± 5.2 *	52.8 ± 6.8 *
	MUFA	11.9 ± 2.0	10.0 ± 2.1	12.0 ± 0.7	14.9 ± 2.0 * ^#^
	PUFA	22.6 ± 5.9	20.0 ± 4.0	31.3 ± 9.0	43.5 ± 6.9 * ^#^
	Total	69.2 ± 10.9	68.0 ± 26.6	94.1 ± 10.6 *	110.7 ± 11.9 * ^#^

The values are expressed in nanomoles per milliliter of urine. * *p* < 0.05—significant difference between CBG, HFHS and HFHS+CBG groups vs. Control group; # *p* < 0.05—significant difference between HFHS+CBG group vs. HFHS group.

## Data Availability

All data generated and analyzed during this study are included in this published article and its Supplementary Information Files.

## Supplementary Materials

The following supporting information can be downloaded at: https://www.mdpi.com/article/10.3390/nu18132063/s1, Tables S1–S8. Cannabigerol (CBG) influence on the fatty acid composition in triacylglycerol (TAG), diacylglycerol (DAG), free fatty acid (FFA), phospholipid (PL) fractions in the kidney tissue (S1–S4) and urine samples (S5–S8) of rats subjected to a standard diet (Control) or a high-fat high-sucrose diet (HFHS).
