# Supplementary material for: Renal Effects of Cannabigerol—Regulation of Lipid Metabolism in the Early Stage of Metabolic Kidney Disorders Induced by High-Fat High-Sucrose Diet
Source: Nutrients. 2026 Jun 24;18(13):2063. doi: 10.3390/nu18132063 (PMC13362918; doi:10.3390/nu18132063)
Supplement: Supplementary file 1 [file nutrients-18-02063-s001.zip › Table S1.pdf]

Table S1. Cannabigerol (CBG) influence on the fatty acids composition in triacylglycerol (TAG) fraction in the kidney tissue of rats subjected to a standard diet (Control) or a high-fat high-sucrose diet (HFHS). The values are expressed in nanomoles per gram of tissue.

		**Control**	**CBG**	**HFHS**	**HFHS+CBG**
SFA	C14:0	4332.5 ± 735.3	2225.1 ± 636.6 *	3126.4 ± 394.6 *	2918.7 ± 645.2
	C16:0	43363.7 ± 11651.3	19284.4 ± 5402.3 *	42671.9 ± 5424.5	37623.9 ± 7848.8
	C18:0	5012.2 ± 1441.4	2583.6 ± 672.6 *	6513.6 ± 1358.8	1810.1 ± 430.6 * #
	C20:0	42.7 ± 11.7	31.4 ± 9.7	46.8 ± 9.3	41.9 ± 11.4
	C22:0	19.5 ± 6.6	19.1 ± 2.7	33.2 ± 11.3	31.8 ± 7.1
	C24:0	13.9 ± 1.3	15.7 ± 2.1	17.1 ± 3.6	15.2 ± 1.9
MUFA	C16:1	14520.9 ± 3368.7	6700.7 ± 1940.2 *	10131.4 ± 864.8 *	8787.7 ± 2185.1 *
	C18:1	32909.0 ± 8764.5	19931.2 ± 2917.2	51710.6 ± 10329.0 *	48989.9 ± 14680.5
	C24:1	12.9 ± 2.7	11.5 ± 1.4	11.6 ± 2.0	12.0 ± 2.1
PUFA	C18:2	10787.6 ± 3084.5	7646.8 ± 2198.3	16592.2 ± 4918.9	13387.9 ± 4098.2
	C18:3	1224.4 ± 308.8	747.9 ± 179.1 *	1155.7 ± 205.5	909.4 ± 279.1

SFA - saturated fatty acid; MUFA - monounsaturated fatty acid; PUFA - polyunsaturated fatty acid; HFHS - high-fat high-sucrose diet; CBG - cannabigerol. *$p<0.05$ – significant difference between CBG, HFHS and HFHS+CBG vs. Control group; #$p<0.05$ – significant difference between HFHS+CBG vs. HFHS group.
