# Supplementary material for: Renal Effects of Cannabigerol—Regulation of Lipid Metabolism in the Early Stage of Metabolic Kidney Disorders Induced by High-Fat High-Sucrose Diet
Source: Nutrients. 2026 Jun 24;18(13):2063. doi: 10.3390/nu18132063 (PMC13362918; doi:10.3390/nu18132063)
Supplement: Supplementary file 1 [file nutrients-18-02063-s001.zip › Table S2.pdf]

**Table S2.** Cannabigerol (CBG) influence on the fatty acids composition in diacylglycerol (DAG) fraction in the kidney tissue of rats subjected to a standard diet (Control) or a high-fat high-sucrose diet (HFHS). The values are expressed in nanomoles per gram of tissue.

| | | **Control** | **CBG** | **HFHS** | **HFHS+CBG** |
|---|---|---|---|---|---|
| SFA | C14:0 | 23.8 ± 6.7 | 21.9 ± 5.6 | 18.4 ± 3.5 | 19.2 ± 3.5 |
| | C16:0 | 341.6 ± 45.2 | 307.5 ± 30.0 | 330.4 ± 49.9 | 305.5 ± 35.5 |
| | C18:0 | 145.1 ± 12.5 | 132.6 ± 11.5 | 134.2 ± 14.8 | 143.0 ± 9.7 |
| | C20:0 | 1.8 ± 0.3 | 2.0 ± 0.3 | 2.1 ± 0.1 | 2.3 ± 0.3 |
| | C22:0 | 0.6 ± 0.0 | 1.0 ± 0.2 * | 1.2 ± 0.2 * | 1.2 ± 0.2 * |
| | C24:0 | 3.2 ± 0.4 | 3.2 ± 0.6 | 3.9 ± 1.1 | 3.8 ± 0.8 |
| MUFA | C16:1 | 38.2 ± 6.9 | 27.0 ± 6.4 * | 30.2 ± 6.0 | 16.5 ± 4.9 * # |
| | C18:1 | 93.1 ± 15.2 | 69.4 ± 14.4 * | 146.0 ± 40.4 * | 103.4 ± 26.3 |
| | C24:1 | 0.8 ± 0.2 | 0.9 ± 0.2 | 1.3 ± 0.4 * | 1.3 ± 0.3 * |
| PUFA | C18:2 | 67.1 ± 12.8 | 49.9 ± 5.9 * | 65.7 ± 14.3 | 60.4 ± 16.9 |
| | C18:3 | 4.5 ± 1.3 | 3.0 ± 0.2 * | 3.7 ± 1.1 | 3.0 ± 0.7 * |

SFA - saturated fatty acid; MUFA - monounsaturated fatty acid; PUFA - polyunsaturated fatty acid; HFHS - high-fat high-sucrose diet; CBG - cannabigerol. *$p$<0.05 – significant difference between CBG, HFHS and HFHS+CBG vs. Control group; #$p$<0.05 – significant difference between HFHS+CBG vs. HFHS group.
