# Supplementary material for: Renal Effects of Cannabigerol—Regulation of Lipid Metabolism in the Early Stage of Metabolic Kidney Disorders Induced by High-Fat High-Sucrose Diet
Source: Nutrients. 2026 Jun 24;18(13):2063. doi: 10.3390/nu18132063 (PMC13362918; doi:10.3390/nu18132063)
Supplement: Supplementary file 1 [file nutrients-18-02063-s001.zip › Table S3.pdf]

Table S3. Cannabigerol (CBG) influence on the fatty acids composition in free fatty acid (FFA) fraction in the kidney tissue of rats subjected to a standard diet (Control) or a high-fat high-sucrose diet (HFHS). The values are expressed in nanomoles per gram of tissue.

		**Control**	**CBG**	**HFHS**	**HFHS+CBG**
SFA	C14:0	10.4 ± 2.5	14.6 ± 2.0 *	5.7 ± 1.1 *	5.1 ± 1.1 *
	C16:0	188.2 ± 42.2	232.7 ± 51.9	174.7 ± 54.0	257.0 ± 39.0 * #
	C18:0	127.5 ± 23.5	114.7 ± 20.8	184.3 ± 29.1 *	166.1 ± 26.5
	C20:0	3.4 ± 0.9	1.8 ± 0.4 *	5.1 ± 1.0 *	2.7 ± 0.6 #
	C22:0	1.9 ± 0.5	0.9 ± 0.2 *	3.6 ± 0.8 *	1.6 ± 0.4 #
	C24:0	18.9 ± 4.1	9.6 ± 1.2 *	32.0 ± 4.6 *	11.1 ± 2.5 * #
MUFA	C16:1	18.5 ± 1.3	11.8 ± 3.6 *	5.0 ± 1.5 *	5.2 ± 1.4 *
	C18:1	96.5 ± 23.6	46.6 ± 10.8 *	46.7 ± 10.6 *	64.9 ± 18.4
	C24:1	1.8 ± 0.5	1.3 ± 0.3	3.4 ± 0.8 *	1.9 ± 0.3 #
PUFA	C18:2	25.3 ± 6.9	25.3 ± 4.5	9.6 ± 1.8 *	18.6 ± 5.1 #
	C18:3	2.4 ± 0.4	2.5 ± 0.4	3.3 ± 0.7	2.0 ± 0.2 #

SFA - saturated fatty acid; MUFA - monounsaturated fatty acid; PUFA - polyunsaturated fatty acid; HFHS - high-fat high-sucrose diet; CBG - cannabigerol. *$p<0.05$ – significant difference between CBG, HFHS and HFHS+CBG vs. Control group; #$p<0.05$ – significant difference between HFHS+CBG vs. HFHS group.
