# Supplementary material for: Renal Effects of Cannabigerol—Regulation of Lipid Metabolism in the Early Stage of Metabolic Kidney Disorders Induced by High-Fat High-Sucrose Diet
Source: Nutrients. 2026 Jun 24;18(13):2063. doi: 10.3390/nu18132063 (PMC13362918; doi:10.3390/nu18132063)
Supplement: Supplementary file 1 [file nutrients-18-02063-s001.zip › Table S4.pdf]

Table S4. Cannabigerol (CBG) influence on the fatty acids composition in phospholipid (PL) fraction in the kidney tissue of rats subjected to a standard diet (Control) or a high-fat high-sucrose diet (HFHS). The values are expressed in nanomoles per gram of tissue.

		**Control**	**CBG**	**HFHS**	**HFHS+CBG**
SFA	C14:0	91.7 ± 10.4	96.0 ± 8.7	117.5 ± 14.8 *	109.6 ± 16.3
	C16:0	11426.7 ± 593.9	11374.5 ± 621.6	10704.4 ± 566.3	11390.3 ± 702.7
	C18:0	12038.2 ± 916.2	12226.8 ± 1032.6	12077.1 ± 813.7	12363.6 ± 716.2
	C20:0	34.6 ± 4.5	36.3 ± 6.7	30.4 ± 5.9	37.1 ± 4.4 #
	C22:0	26.8 ± 4.7	23.7 ± 5.9	29.8 ± 4.1	25.2 ± 5.3
	C24:0	37.8 ± 4.7	31.6 ± 8.1	34.7 ± 9.9	22.3 ± 3.8 * #
MUFA	C16:1	536.9 ± 69.1	515.2 ± 104.4	303.5 ± 60.9 *	319.8 ± 45.2 *
	C18:1	3474.1 ± 337.8	3498.4 ± 158.1	3873.7 ± 159.4 *	4234.0 ± 286.6 * #
	C24:1	30.1 ± 5.8	28.7± 11.6	27.3 ± 2.1	28.9 ± 3.4
PUFA	C18:2	7461.7 ± 658.6	7566.7 ± 782.0	6022.3 ± 586.4 *	6666.5 ± 608.0
	C18:3	74.0 ± 8.7	70.1 ± 6.6	48.4 ± 5.9 *	57.3 ± 11.8 *

SFA - saturated fatty acid; MUFA - monounsaturated fatty acid; PUFA - polyunsaturated fatty acid; HFHS - high-fat high-sucrose diet; CBG - cannabigerol. *$p<0.05$ – significant difference between CBG, HFHS and HFHS+CBG vs. Control group; #$p<0.05$ – significant difference between HFHS+CBG vs. HFHS group.
