# Supplementary material for: Renal Effects of Cannabigerol—Regulation of Lipid Metabolism in the Early Stage of Metabolic Kidney Disorders Induced by High-Fat High-Sucrose Diet
Source: Nutrients. 2026 Jun 24;18(13):2063. doi: 10.3390/nu18132063 (PMC13362918; doi:10.3390/nu18132063)
Supplement: Supplementary file 1 [file nutrients-18-02063-s001.zip › Table S5.pdf]

Table S5. Cannabigerol (CBG) influence on the fatty acids composition in triacylglycerol (TAG) fraction in urine samples of rats subjected to a standard diet (Control) or a high-fat high-sucrose diet (HFHS). The values are expressed in nanomoles per milliliter of urine.

		**Control**	**CBG**	**HFHS**	**HFHS+CBG**
SFA	C14:0	2.5 ± 0.3	2.1 ± 0.4	4.1 ± 0.8 *	2.6 ± 0.6 #
	C16:0	18.5 ± 3.7	14.6 ± 2.8	23.5 ± 6.0	18.0 ± 4.2
	C18:0	3.1 ± 0.8	3.1 ± 0.4	7.8 ± 1.6 *	4.9 ± 1.3 * #
	C20:0	0.6 ± 0.2	0.6 ± 0.1	0.7 ± 0.2	0.4 ± 0.1 * #
	C22:0	0.8 ± 0.2	0.9 ± 0.2	1.0 ± 0.2	0.6 ± 0.1 #
	C24:0	0.6 ± 0.2	0.9 ± 0.2 *	0.7 ± 0.2	0.4 ± 0.1 #
MUFA	C16:1	3.4 ± 0.7	2.8 ± 0.4	3.6 ± 0.6	2.2 ± 0.3 * #
	C18:1	33.1 ± 5.2	23.0 ± 5.0 *	36.4 ± 10.1	24.9 ± 5.5 * #
	C24:1	0.7 ± 0.0	0.8 ± 0.2	0.7 ± 0.2	0.4 ± 0.1 * #
PUFA	C18:2	11.5 ± 2.7	6.9 ± 1.7 *	8.5 ± 0.8 *	6.4 ± 1.2 * #
	C18:3	1.8 ± 0.1	1.8 ± 0.3	1.7 ± 0.3	1.1 ± 0.3 * #
	C20:4	0.6 ± 0.1	0.7 ± 0.1	0.7 ± 0.2	0.4 ± 0.1 * #
	C20:5	0.9 ± 0.2	0.8 ± 0.2	0.7 ± 0.2	0.5 ± 0.0
	C22:6	0.5 ± 0.1	0.9 ± 0.2 *	0.7 ± 0.2 *	0.1 ± 0.1 * #

SFA - saturated fatty acid; MUFA - monounsaturated fatty acid; PUFA - polyunsaturated fatty acid; HFHS - high-fat high-sucrose diet; CBG - cannabigerol. *$p<0.05$ – significant difference between CBG, HFHS and HFHS+CBG vs. Control group; #$p<0.05$ – significant difference between HFHS+CBG vs. HFHS group.
