# Supplementary material for: Renal Effects of Cannabigerol—Regulation of Lipid Metabolism in the Early Stage of Metabolic Kidney Disorders Induced by High-Fat High-Sucrose Diet
Source: Nutrients. 2026 Jun 24;18(13):2063. doi: 10.3390/nu18132063 (PMC13362918; doi:10.3390/nu18132063)
Supplement: Supplementary file 1 [file nutrients-18-02063-s001.zip › Table S6.pdf]

Table S6. Cannabigerol (CBG) influence on the fatty acids composition in diacylglycerol (DAG) fraction in urine samples of rats subjected to a standard diet (Control) or a high-fat high-sucrose diet (HFHS). The values are expressed in nanomoles per milliliter of urine.

		**Control**	**CBG**	**HFHS**	**HFHS+CBG**
SFA	C14:0	2.8 ± 0.6	1.7 ± 0.4 *	2.9 ± 0.6	1.9 ± 0.6 #
	C16:0	20.4 ± 5.1	13.9 ± 2.3 *	20.0 ± 2.6	12.9 ± 2.4 * #
	C18:0	9.1 ± 2.2	6.8 ± 1.3	9.1 ± 2.4	6.9 ± 1.2
	C20:0	0.4 ± 0.1	0.3 ± 0.1	0.4 ± 0.1	0.2 ± 0.0 * #
	C22:0	0.1 ± 0.0	0.2 ± 0.0	0.1 ± 0.0	0.1 ± 0.0
	C24:0	0.2 ± 0.0	0.2 ± 0.0	0.2 ± 0.0	0.1 ± 0.0 * #
MUFA	C16:1	0.9 ± 0.2	0.4 ± 0.1 *	0.4 ± 0.1 *	0.3 ± 0.1 *
	C18:1	15.7 ± 1.5	5.1 ± 1.5 *	4.6 ± 1.1 *	3.0 ± 0.6 * #
	C24:1	n/a	n/a	n/a	n/a
PUFA	C18:2	4.0 ± 0.6	1.7 ± 0.4 *	1.4 ± 0.3 *	1.1 ± 0.3 *
	C18:3	0.4 ± 0.1	0.3 ± 0.1	0.2 ± 0.0 *	0.2 ± 0.0 *
	C20:4	0.3 ± 0.1	0.3 ± 0.0	0.4 ± 0.1	0.7 ± 0.1 * #
	C20:5	n/a	n/a	n/a	n/a
	C22:6	n/a	n/a	n/a	n/a

SFA - saturated fatty acid; MUFA - monounsaturated fatty acid; PUFA - polyunsaturated fatty acid; HFHS - high-fat high-sucrose diet; CBG - cannabigerol. *p<0.05 – significant difference between CBG, HFHS and HFHS+CBG vs. Control group; #p<0.05 – significant difference between HFHS+CBG vs. HFHS group.
