# Supplementary material for: Renal Effects of Cannabigerol—Regulation of Lipid Metabolism in the Early Stage of Metabolic Kidney Disorders Induced by High-Fat High-Sucrose Diet
Source: Nutrients. 2026 Jun 24;18(13):2063. doi: 10.3390/nu18132063 (PMC13362918; doi:10.3390/nu18132063)
Supplement: Supplementary file 1 [file nutrients-18-02063-s001.zip › Table S7.pdf]

Table S7. Cannabigerol (CBG) influence on the fatty acids composition in free fatty acid (FFA) fraction in urine samples of rats subjected to a standard diet (Control) or a high-fat high-sucrose diet (HFHS). The values are expressed in nanomoles per milliliter of urine.

		Control	CBG	HFHS	HFHS+CBG
SFA	C14:0	1.1 ± 0.2	0.8 ± 0.2	1.4 ± 0.4	1.4 ± 0.3
	C16:0	9.0 ± 1.9	6.6 ± 1.5 *	14.9 ± 0.6 *	13.5 ± 1.9 *
	C18:0	4.5 ± 1.0	3.4 ± 0.9	8.1 ± 0.4 *	7.3 ± 1.4 *
	C20:0	0.1 ± 0.0	0.2 ± 0.0 *	0.2 ± 0.0	0.2 ± 0.0 *
	C22:0	0.1 ± 0.0	0.1 ± 0.0	0.1 ± 0.0	0.1 ± 0.0
	C24:0	0.1 ± 0.0	0.1 ± 0.0	0.1 ± 0.0 *	0.1 ± 0.0
MUFA	C16:1	0.3 ± 0.1	0.3 ± 0.1	0.2 ± 0.0 *	0.2 ± 0.0 *
	C18:1	1.6 ± 0.3	1.1 ± 0.3	2.1 ± 0.4 *	2.2 ± 0.2 *
	C24:1	n/a	n/a	n/a	n/a
PUFA	C18:2	0.6 ± 0.2	0.4 ± 0.1 *	0.6 ± 0.1	0.5 ± 0.1
	C18:3	0.1 ± 0.0	0.2 ± 0.0	0.1 ± 0.0	0.1 ± 0.0
	C20:4	0.1 ± 0.0	0.2 ± 0.0	0.1 ± 0.0	0.1 ± 0.0
	C20:5	n/a	n/a	n/a	n/a
	C22:6	n/a	n/a	n/a	n/a

SFA - saturated fatty acid; MUFA - monounsaturated fatty acid; PUFA - polyunsaturated fatty acid; HFHS - high-fat high-sucrose diet; CBG - cannabigerol. *$p<0.05$ – significant difference between CBG, HFHS and HFHS+CBG vs. Control group; #$p<0.05$ – significant difference between HFHS+CBG vs. HFHS group.
