# Supplementary material for: Renal Effects of Cannabigerol—Regulation of Lipid Metabolism in the Early Stage of Metabolic Kidney Disorders Induced by High-Fat High-Sucrose Diet
Source: Nutrients. 2026 Jun 24;18(13):2063. doi: 10.3390/nu18132063 (PMC13362918; doi:10.3390/nu18132063)
Supplement: Supplementary file 1 [file nutrients-18-02063-s001.zip › Table S8.pdf]

Table S8. Cannabigerol (CBG) influence on the fatty acids composition in phospholipid (PL) fraction in urine samples of rats subjected to a standard diet (Control) or a high-fat high-sucrose diet (HFHS). The values are expressed in nanomoles per milliliter of urine.

		**Control**	**CBG**	**HFHS**	**HFHS+CBG**
SFA	C14:0	1.9 ± 0.3	2.0 ± 0.3	2.0 ± 0.4	2.4 ± 0.5
	C16:0	14.9 ± 3.1	11.6 ± 1.9	18.5 ± 3.5	21.3 ± 3.7 *
	C18:0	14.0 ± 2.7	11.7 ± 2.6	21.7 ± 4.5 *	26.8 ± 3.2 *
	C20:0	0.8 ± 0.1	0.8 ± 0.2	1.1 ± 0.2 *	0.8 ± 0.2
	C22:0	0.8 ± 0.2	1.0 ± 0.1	1.2 ± 0.2 *	1.2 ± 0.3
	C24:0	1.3 ± 0.2	1.3 ± 0.2	1.6 ± 0.2 *	1.4 ± 0.3
MUFA	C16:1	1.6 ± 0.2	1.7 ± 0.2	2.3 ± 0.3 *	2.1 ± 0.2 *
	C18:1	9.0 ± 1.9	6.8 ± 1.7	8.6 ± 0.3	12.1 ± 1.6 * #
	C24:1	1.3 ± 0.3	1.3 ± 0.2	1.5 ± 0.3	1.3 ± 0.2
PUFA	C18:2	5.6 ± 1.0	4.8 ± 1.4	5.9 ± 1.2	7.6 ± 1.8 #
	C18:3	0.7 ± 0.2	0.8 ± 0.1	1.0 ± 0.1 *	0.9 ± 0.2
	C20:4	13.5 ± 3.8	9.4 ± 2.3	19.3 ± 4.7	30.6 ± 6.5 * #
	C20:5	1.4 ± 0.3	1.4 ± 0.3	1.6 ± 0.4	1.1 ± 0.3 #
	C22:6	3.0 ± 0.3	3.2 ± 0.6	3.6 ± 0.3 *	3.9 ± 0.6 *

SFA - saturated fatty acid; MUFA - monounsaturated fatty acid; PUFA - polyunsaturated fatty acid; HFHS - high-fat high-sucrose diet; CBG - cannabigerol. *p<0.05 – significant difference between CBG, HFHS and HFHS+CBG vs. Control group; #p<0.05 – significant difference between HFHS+CBG vs. HFHS group.
